# The Role of Cognitive Control in Age-Related Changes in Well-Being

**DOI:** 10.3389/fnagi.2020.00198

**Published:** 2020-07-09

**Authors:** Ayano Yagi, Rui Nouchi, Kou Murayama, Michiko Sakaki, Ryuta Kawashima

**Affiliations:** ^1^Research Institute, Kochi University of Technology, Kami, Japan; ^2^Institute of Development, Aging, and Cancer (IDAC), Tohoku University, Sendai, Japan; ^3^School of Psychology and Clinical Language Sciences, University of Reading, Reading, United Kingdom

**Keywords:** socioemotional selectivity theory, cognitive control, executive functioning, Eastern Asia, subjective well-being, psychological well-being

## Abstract

Maintaining emotional well-being in late life is crucial for achieving successful and healthy aging. While previous research from Western cultures has documented that emotional well-being improves as individuals get older, previous research provided mixed evidence on the effects of age on well-being in Eastern Asian cultures. However, previous studies in East Asia do not always take into account the effects of cognitive control—an ability which has been considered as a key to enable older adults to regulate their emotions. In the current study, we tested whether cognitive control abilities interact with age in determining individuals’ well-being in 59 Japanese females (age range: 26–79; *M*_age_ = 64.95). Participants’ mental health and mental fatigue were tracked for 5 years together with their cognitive control abilities. We found that as individuals became older, they showed improved mental health and decreased mental fatigue. In addition, we found a quadratic effect of age on mental fatigue, which was further qualified by baseline cognitive control abilities. Specifically, in those who had a lower level of cognitive control abilities, mental fatigue declined until the mid-60s, at which point it started increasing (a U-shape effect). In contrast, in those who had a higher level of cognitive control ability, mental fatigue showed a steady decrease with age even after their mid-60s. These results suggest that whether advancing age is associated with positive vs. negative changes in well-being depends on cognitive control abilities, and that preserved cognitive control is a key to maintain well-being in late life.

## Introduction

Aging is typically associated with a range of negative experiences, such as declines in physical functioning and a loss of close friends. Nevertheless, when older adults are invited to lab experiments, they are more likely to pay attention to and remember positive information than negative information (Mather and Knight, 2005; Knight et al., 2007; Sakaki et al., 2013; Reed et al., 2014). This age-by-valence interaction has been called the positivity effect. In addition to the positivity effect in memory and attention, older adults also report better emotional experiences, reduced negative emotions, and improved well-being than do younger adults (Charles et al., 2001, 2010; Riediger et al., 2009; Carstensen et al., 2011; Steptoe et al., 2015). A prominent theory to explain the positivity effect and better well-being with age is the socioemotional selectivity theory (SST; Scheibe and Carstensen, 2010). The SST posits that as individuals get older, they perceive time left in their life as being more limited. As a result, older adults are more likely to prioritize emotion regulation goals and invest more effort in what is most important for their emotional well-being than do younger adults. This motivational shift is considered to result in preferential processing of positive stimuli as well as better emotional experiences in old age.

According to the SST, the positivity effect and better well-being with age should be driven by one’s perception of the limited time left in their life. Given that chronological age is typically associated with a limited time perspective across many cultures (e.g., Lang and Carstensen, 2002; Fung and Carstensen, 2006), it is plausible that the positivity effect and better well-being with age are observed irrespective of cultures. However, previous research does not always support this prediction, and evidence from Eastern Asia is rather mixed (for a review see Fung, 2013). For example, while American individuals show increased dispositional optimism with age, Chinese individuals show decreased dispositional optimism with age (You et al., 2009). A recent large-scale cross-sectional study also reveals a smaller age-related improvement in emotional experiences in Japan than in the US (Grossmann et al., 2014). In addition, studies on attention and memory failed to observe the positivity effect in East Asian cultures (e.g., Fung et al., 2010). For example, while older adults in Western cultures typically show selective attention to positive stimuli over negative stimuli (Mather and Carstensen, 2003), Chinese older adults looked away from positive stimuli (Fung et al., 2008a).

In contrast, other studies suggest that the age-related positivity effect is not limited to Western cultures and can be observed in Eastern Asian cultures (Fung and Carstensen, 2006). When Korean participants were compared to US participants, Korean older adults show a similar age-related positivity effect to US older adults in memory tasks (Kwon et al., 2009; Ko et al., 2011). A recent study also reveals that Chinese older adults demonstrate larger preferences toward positive stimuli in attention (Wang et al., 2015) as observed in US older adults (Knight et al., 2007).

This inconsistent pattern of results may be driven by individual differences in factors relevant to the positivity effect and well-being (Fung et al., 2010). One possible source of individual differences in cognitive control (Mather and Carstensen, 2005). Cognitive control is critical for achieving emotion regulation (Urry and Gross, 2010; Opitz et al., 2012) but it is one of the most vulnerable processes of age-related decline (e.g., Milham et al., 2002; Fjell et al., 2016). Research from Western cultures suggests that this age-related decline in the cognitive control mechanisms can lead to weaker positivity effects and worse well-being in the late old age. For example, the positivity effect in memory and attention is attenuated when older adults have limited resources in their executive functioning (Mather and Knight, 2005; Knight et al., 2007; Petrican et al., 2008). Individual differences in the positivity effect in memory were also correlated with individual differences in cognitive control (Mather and Knight, 2005; Sakaki et al., 2019).

Previous research on well-being in old age further suggests that preserved cognitive function, including cognitive control abilities, plays key roles in older adults’ well-being. For example, cross-sectional studies demonstrated that higher general intelligence is associated with better emotional experiences in older adults (Isaacowitz and Smith, 2003; Gale et al., 2012). Higher cognitive control abilities are also correlated with higher scores in the purpose of life (Lewis et al., 2017)—one of the key domains of psychological well-being (Ryff, 1989). Longitudinal studies also show that better processing speed at baseline is associated with subsequent better life satisfaction (Enkvist et al., 2013). Recent research further extends these findings and indicates that cognitive decline predicts the subsequent level of well-being in older adults (Wilson et al., 2013; Allerhand et al., 2014; Godin et al., 2018).

Despite its importance, most of the previous studies on the positivity effect and well-being in East Asian cultures did not consider the effects of cognitive control. The current study aims to address the role of cognitive control in longitudinal changes in well-being in Japanese participants. We tracked Japanese individuals’ well-being and cognitive control performance for up to 5 years, used growth curve modeling, and tested whether the baseline cognitive control performance is critical for individuals to show the age-related improvements in well-being. We hypothesized that: (a) individuals’ well-being improves as they get older; but (b) this effect of age is qualified by the baseline cognitive control abilities, such that those who are low in their cognitive control abilities show smaller improvements in their well-being with age especially in the late adulthood when their cognitive control mechanisms start declining.

## Materials and Methods

### Participants

Fifty-nine Japanese females participated in the study. Four participants did not complete the digit backward task and another participant did not complete the digit backward task and the Stroop task. Given that these tasks were used to estimate cognitive control abilities, data from the five participants were excluded; thus analyses were performed on data from 54 participants (age range at Wave 1: 26–79; *M*_age_ = 64.95, *SD* = 11.08). Participants were recruited through advertisements in local newspapers during July 2011, from Sendai city around the Tohoku University, Japan. We recruited as many participants as possible during this recruitment period. Data were collected 12 times between August 2011 and June 2016 (Wave 1: August 2011; Wave 2: September 2011; Wave 3: November 2011; Wave 4: February 2012; Wave 5: September 2012; Wave 6: February 2013; Wave 7: August 2013; Wave 8: February 2014; Wave 9: August 2014; Wave 10: March 2015; Wave 11: September 2015; Wave 12: June 2016; a total number of data points = 382). None of the participants had a history of the following mental disorders including schizophrenia, bipolar disorder, major depressive disorder, anxiety disorders, obsessive-compulsive disorder and disease known to affect the central nervous system, such as thyroid disease, multiple sclerosis, Parkinson disease, stroke, severe hypertension (systolic blood pressure over 180, diastolic blood pressure over 110), and diabetes. None of them took drugs that affected cognitive function (including benzodiazepines, antidepressants, and other central nervous agents). None of them reported as being diagnosed as dementia or MCI (mild cognitive impairment). They received 1,000 JPY (approx. $10)/hour for their participation.

### Instruments

We used a Japanese version of the subjective well-being inventory (SUBI) which consists of two subscales of well-being (Sell and Nagpal, 1992); (a) 19 items on mental health: (e.g., “Do you feel your life is interesting?”; “Do you normally accomplish what you want to?”); and (b) 21 items on mental fatigue (e.g., “Do you feel disturbed by feelings of anxiety and tension?”; “Do you sometimes feel sad without reason?”). Participants responded to each question on a scale of 1 (“not so much”), 2 (“somewhat agree”); and 3 (“strongly agree”). The reliability and validity of the Japanese version were reported in a previous study (Ono and Yoshimura, 2001). Higher mental health scores indicate a higher degree of subjective health and higher mental fatigue scores indicate a higher degree of mental fatigue (i.e., low subjective well-being).

### Procedure

The data analyzed in this article were collected as a part of a more extensive study (see Supplementary Materials for other measures we obtained). Participants were told that the study aimed to examine the longitudinal changes in women’s mental functioning. For each time point, a paper-based questionnaire packet including SUBI was mailed to participants and completed at home. Participants next completed a lab session, where we administered the article paper-pencil Stroop task (Hakoda and Sasaki, 1990; Takeuchi et al., 2011), the Japanese Adult Reading Test (JART; Matsuoka et al., 2006), verbal memory using a short-paragraph story (Wechsler, 1987), the digit forward/backward task from the WAIS (Wechsler, 1997) in groups (which included 1–6 participants; see Supplementary Materials for details).

### Data Analysis

The primary goal of this study was to test the role of cognitive control abilities. Therefore, we focused on Stroop and Digit Backward performance as a measure of individuals’ cognitive control ability at Wave 1 and created a composite score based on performance in these two tasks; A similar measure was computed in previous studies as well (Davis et al., 2010). The Stroop interference score was reversed so that a higher score reflects a better cognitive control ability. We then created a single cognitive control index by averaging the z-scores of the Stroop interference score and backward digit span task, *r*_(52)_ = 0.45, *p* < 0.001.

While none of our participants reported as being clinically diagnosed with dementia nor MCI, we ran another analysis to account for the effects of possible dementia/MCI cases. Specifically, we created another composite measure based on the Wechsler logical memory and vocabulary performance, both of which is typically correlated with performance in standard screening batteries for dementia (Raghavan et al., 2013; Chapman et al., 2016). This additional composite score was used as a covariate in our supplementary analyses to rule out the possibility that the effects of cognitive control abilities are driven by general cognitive declines associated with dementia or MCI (see Supplementary Results); the correlation between the two composite scores was *r*_(48)_ = 0.37, *p* < 0.001.

## Results

Table 1 reports the descriptive statistics of the main variables for each time point. The attrition rate for the second wave was 3.70%, increased over time, and was 74.07% at Wave 12 (Table 1). We compared the variables reported in this article at Wave 1 between participants who participated all of the assessments and those who did not, but did not observe any significant difference between the groups; SUBI Mental Health, *t*_(24)_ = 2.06, *p* = 0.785, *d* = 0.08, SUBI Mental Fatigue *t*_(18)_ = 2.10, *p* = 0.123, *d* = 0.54, and the cognitive control index, *t*_(23)_ = 2.07, *p* = 0.373, *d* = 0.28.

**Table 1:** Descriptives statistics of key variables reported in the manuscript.

	SUBI mental health			SUBI mental fatigue			Interference rates of stroop task		Backward recall task	
	*M* (*SD*)	*α*	*N*	*M* (*SD*)	*α*	*N*	*M* (SD)	*N*	*M* (*SD*)	*N*
Wave 1	37.28 (5.21)	0.84	54	32.92 (5.59)	0.83	54	16.65 (17.23)	54	6.59 (2.14)	54
Wave 2	37.15 (5.84)	0.88	52	32.25 (5.53)	0.82	52	15.26 (15.84)	51	6.46 (2.31)	50
Wave 3	38.43 (4.87)	0.79	48	31.71 (5.10)	0.80	48	12.69 (16.73)	46	6.91 (2.58)	46
Wave 4	37.47 (4.31)	0.76	44	31.25 (5.03)	0.82	43	11.46 (9.41)	44	6.98 (2.76)	43
Wave 5	38.91 (5.30)	0.84	36	31.63 (5.10)	0.80	36	8.51 (12.83)	36	6.92 (2.75)	36
Wave 6	38.03 (4.21)	0.80	32	30.64 (4.01)	0.73	32	11.09 (10.97)	32	6.53 (2.72)	32
Wave 7	39.09 (5.41)	0.87	27	31.37 (4.82)	0.81	27	12.28 (12.73)	26	6.54 (2.42)	26
Wave 8	39.38 (6.92)	0.92	20	31.36 (4.13)	0.71	20	16.59 (11.33)	20	6.65 (2.72)	20
Wave 9	38.97 (6.83)	0.91	19	31.35 (4.92)	0.81	19	14.70 (8.90)	19	7.00 (2.11)	19
Wave 10	37.70 (5.91)	0.88	19	32.29 (4.27)	0.69	19	14.03 (9.22)	17	6.53 (2.48)	17
Wave 11	37.78 (5.74)	0.88	17	30.83 (4.39)	0.76	17	14.31 (17.50)	17	7.18 (2.51)	17
Wave 12	38.30 (6.85)	0.93	14	32.32 (4.37)	0.72	14	9.87 (11.77)	14	6.57 (2.44)	14

*Notes: α shows consistency within the scale*.

### Growth Curve Analysis of Well-Being

We next tested our main prediction that baseline cognitive control abilities predict age-related changes in well-being. To test this prediction, we used a growth curve model on SUBI mental health and SUBI mental fatigue scores (they were modeled separately) with cognitive control assessed at Wave 1 as a predictor. The model was specified and estimated by hierarchical linear modeling, using the software R version 3.6.0 (R-Core-Team, 2017) and the “lme4” package (version 1.1–21), with Waves as level-1 units and participants as level-2 units. To examine how well-being changed as a function of participants’ age, the model included the linear and quadratic effects of age as level-1 predictors. The model also included the random intercept and random slopes of the linear and the quadratic effects of age. The model is as follows:

$$\begin{array}{c} \text{Level-1}:{\text{y}}_{\text{ij}}\text{=}\beta_{\text{0j}}\text{+}\beta_{\text{1j}}\left({\text{Age}}_{\text{ij}}\right)\text{+}\beta_{\text{2j}}{\left({\text{Age}}_{\text{ij}}\right)}^{\text{2}}\text{+}r_{\text{ij}}\\ \text{Level-2}:\beta_{\text{0j}}\text{=}\gamma_{\text{00}}\text{+}\gamma_{\text{01}}\left(\text{Cognitive }{\text{control}}_{\text{j}}\right)\text{+}u_{\text{0j}}\\ \beta_{\text{1}j}\text{=}\gamma_{\text{10}}\text{+}\gamma_{\text{11}}\left(\text{Cognitive }{\text{control}}_{\text{j}}\right)\text{+}u_{\text{1j}}\\ \beta_{\text{2}j}\text{=}\gamma_{\text{20}}\text{+}\gamma_{\text{21}}\left(\text{Cognitive }{\text{control}}_{\text{j}}\right)\text{+}u_{\text{2j}} \end{array}$$

*y*_ij_ was the well-being (mental health or mental fatigue scores) of the participant *j* at Wave *i*. Age_ij_ is the age of participant *j* at Wave *i* (to facilitate interpretation and computation, we divided the actual age by 10), with grand-mean centered. *β*_0j_ was the intercept of participant *j* (the estimated value of the dependent variable at Wave 1), whereas *β*_1j_ and *β*_2j_ represent the linear and quadratic effects of age for participant *j*. Cognitive control_j_ is the cognitive control score of participant *j* at Wave 1, and this variable was included to explain individual differences in the intercept and slopes (*β*_0j_, *β*_1j_, and *β*_2j_). *u*_0j_*, u*_1j_, and *u*_2j_ refer to the random intercept and random slopes between participants which were unexplained by the cognitive control scores. *r*_ij_ is the error term following a normal distribution. Given that the model described above did not converge when the dependent variable was a mental health score, we removed the random slope for the quadratic effects of age for the mental health score.

**Figure 1:**
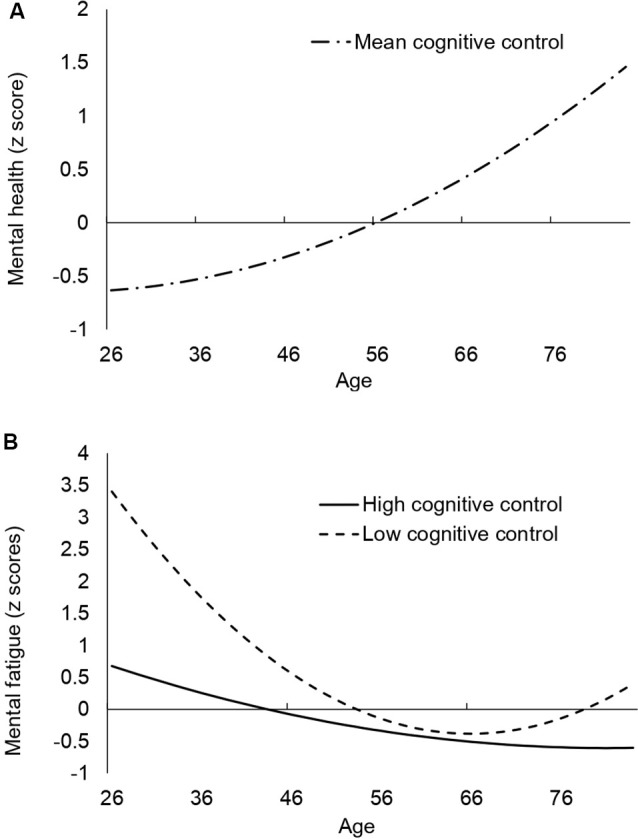
**(A)** Estimation of the growth curve of mental health. The line represents the mental health score when entering the mean cognitive control score. **(B)** Estimation of the growth curve of mental fatigue. The solid line represents higher cognitive control abilities at Wave 1; and the dashed line represents lower cognitive control abilities at Wave 1 (±1 SD than the mean, respectively).

Consistent with the age-related improvement in well-being documented in the literature, we found the positive effects of age on mental health scores (Table 2), *γ*_10_ = 0.361, *p* < 0.05, indicating that participants’ mental health improved as they became older. The quadratic effects of age, cognitive control, and any interactions between age and cognitive control were not significant (Figure 1A).

**Table 2:** Results from multilevel growth curve analyses on two aspects of subjective well-being.

	Mental health		Mental fatigue	
Fixed effects	Coefficient	*p*	Coefficient	*p*
Intercept	−0.03	0.841	−0.21	0.124
Cognitive control	0.04	0.813	−0.13	0.434
Age	0.36*	0.021	−0.37*	0.001
Age*Cognitive control	−0.31	0.092	0.18	0.138
Age^2^	0.05	0.571	0.14*	0.007
Age^2^*Cognitive control	−0.03	0.730	−0.12*	0.013
**Random effects**	**Variance**		**Variance**
Intercept	0.73		0.77
Age	0.15		0.01
Age^2^	NA		0.01

Note: Age^2^ stands for the quadratic effects of age. *Significant effects.

A similar analysis on mental fatigue also revealed a significant effect of age (Table 2), *γ*_10_ = -0.369, *p* = 0.001, which indicates less mental fatigue (i.e., better well-being) as participants became older, again supporting the idea of better well-being with age. In addition, we found a significant positive quadratic effect of age, *γ*_20_ = 0.140, *p* < 0.01, which was further moderated by cognitive control at Wave 1, *γ*_21_ = -0.116, *p* < 0.05. Figure 1B plotted the growth curve for those with high (mean +1 SD) and low (mean −1 SD) cognitive control scores at Wave 1. Participants with lower cognitive control levels at Wave 1 showed the U-shape effects of age, suggesting that their well-being improved until mid-60 at which point it started decreasing. These results are consistent with previous results that the age-related improvements in well-being stop emerging after a certain point (Carstensen et al., 2011; Jivraj et al., 2014; Gana et al., 2015). In contrast, individuals with higher levels of cognitive control at Wave 1 showed a linear positive effect of age, where increasing age led to less mental fatigue (i.e., greater subjective well-being) even after the age of 65. In both mental fatigue and mental health, the results remained unchanged when including the additional covariate based on vocabulary and memory performance (see Supplementary Table S1).

### Growth-Curve Analysis of Cognitive Control

While our primary focus is on the effects of cognitive control on age-related changes in well-being, previous research shows that well-being also protects against age-related changes in cognitive function (Wilson et al., 2013; Allerhand et al., 2014). To test this possibility, we examined whether and how the well-being assessed at Wave 1 affects the growth curve of cognitive control. The specified growth-curve model was similar to the one used in the previous section but now the dependent variable was the cognitive control scores. Mental health and mental fatigue scores at Wave 1 were included simultaneously as level-2 predictors to explain the individual differences in the linear and quadratic effects of age (see below).

$$\begin{array}{c} \text{Level-1: }yij=\beta_{0\text{j}}+\beta_{1\text{j}}({\text{Age}}_{\text{ij}})+\beta_{2\text{j}}{\left({\text{Age}}_{\text{ij}}\right)}^{2}+{\text{r}}_{\text{ij}}\\ \text{Level-2: }\beta_{0\text{j}}=\gamma_{00}+\gamma_{01}({\text{mental health}}_{\text{j}})+\gamma_{02}({\text{mental fatigue}}_{\text{j}})+u\\ \beta_{1\text{j}}=\gamma_{10}+\gamma_{11}({\text{mental fatigue}}_{j})+\gamma_{12}({\text{mental fatigue}}_{j})+u_{1j}\\ \beta_{2\text{j}}=\gamma_{20}+\gamma_{21}({\text{mental health}}_{j})+\gamma_{22}({\text{mental fatigue}}_{j})+u_{2\text{j}} \end{array}$$

This analysis revealed that neither of the mental health measures at Wave 1 predicted age-related changes in cognitive control (*p*s = 0.540, 0.273; Table 3). However, we found a significant linear effect of age, indicating that as individuals get older, their cognitive control ability declines.

**Table 3:** Results from multilevel growth curve analyses on cognitive control performance.

Fixed effects	Coefficient	*p*
Intercept	0.074	0.442
Mental health (wave 1)	−0.072	0.540
Mental fatigue (wave 1)	−0.130	0.273
Age	−0.358*	0.002
Age*Mental health	−0.092	0.411
Age*Mental fatigue	−0.205	0.123
Age^2^	0.005	0.937
Age^2^*Mental health	0.004	0.953
Age^2^*Mental fatigue	−0.071	0.277
**Random effects**	**Variance**	
Intercept	0.307
Age	0.061
Age^2^	0.212

Note: Age^2^ stands for the quadratic effects of age. *Significant effects.

## Discussion

Well-being in old age has been associated with a better survival rate and lower risks to various diseases (Steptoe and Wardle, 2011; Boehm and Kubzansky, 2012), suggesting that it is key for achieving successful aging. Research done in Western cultures often demonstrate that as individuals get older, they preferentially process emotionally positive information (Mather and Knight, 2005; Knight et al., 2007; Sakaki et al., 2013; Reed et al., 2014) and report better well-being (Charles et al., 2001, 2010; Riediger et al., 2009; Carstensen et al., 2011; Steptoe et al., 2015). In contrast, studies in Eastern Asian cultures have provided mixed evidence (Fung et al., 2008a, 2010; Grossmann et al., 2014). Yet many of these studies done in East Asian culture did not consider individual differences in cognitive control—an ability which has been considered to play a key role in the age-related positivity effect and wellbeing in older adults.

In the current study, longitudinal data on well-being and cognitive control abilities from Japanese females were analyzed to address: (a) whether age has positive impacts on well-being as observed in Western culture; and (b) whether such effects of age on well-being are modulated by the levels of cognitive control. We measured two aspects of subjective well-being (one on mental health and another on mental fatigue) and found that advancing age is associated with increased levels of mental health and decreased levels of mental fatigue, replicating the age-related increases in well-being in this sample. In addition to the main effects of age, the analysis of the mental fatigue score revealed that age has quadratic effects on mental fatigue that were further qualified by the baseline cognitive control ability. Specifically, in those who had lower levels of cognitive control ability at baseline, advancing age stopped having positive impacts on well-being after the mid-60s as observed in previous studies (Carstensen et al., 2011; Jivraj et al., 2014; Gana et al., 2015). In contrast, those who had higher levels of cognitive control showed a steady decrease in their mental fatigue level even after their mid-60s. These results support findings in previous studies from Western cultures and suggest that cognitive control plays key roles in protecting subjective well-being when individuals get older even in East Asia.

Our results support the SST and suggest that the effects of age on well-being are similar across Western and East Asian cultures. However, it does not mean that individuals achieve better well-being in a similar manner across different cultures. Past studies suggest that people from different cultures often take different behaviors to achieve better well-being (Tsai, 2017). For example, in East Asian cultures that are more collectivistic, people tend to focus more on interpersonal relatedness to achieve better well-being than those in Western cultures (Fung et al., 2008b). Furthermore, unlike Westerners who value high arousal positive states, East Asians value low arousal positive states. They, therefore, take behavior that induces low arousal positive emotions to achieve better well-being (Tsai et al., 2007). Future research needs to further examine the interaction between age and cultures on strategies/behavior people take to achieve better well-being.

In addition to the effects of age on well-being, we also examined the effects of age on cognitive control abilities and confirmed the previous findings that cognitive control abilities decline as individuals get older as documented in the literature (e.g., Milham et al., 2002; Tucker-Drob, 2011; Fjell et al., 2016). However, while previous research demonstrates that well-being helps protect against age-related changes in cognitive function (Wilson et al., 2013; Allerhand et al., 2014), we did not find significant effects of well-being on cognitive control abilities. The lack of significant effects of well-being may be driven by relatively small sample size in this study. Future studies need to use a larger sample to examine the interaction between well-being and cognitive control abilities.

Another question for future research concerns similarities and differences in the mechanisms underlying the age-related increases in well-being vs. the age-related positivity effect in attention and memory. Previous research has documented that as individuals get older, they show improved well-being as well as more preferences for positive over negative materials in attention and memory. Both these two phenomena have been explained by the SST (Steptoe et al., 2015; Carstensen and DeLiema, 2018). Our results support these notions and suggest that the effects of age on well-being and emotional processing are at least partly explained by the same factor—cognitive control abilities. However, there should be other factors that are uniquely associated with each of them. Individuals’ well-being is affected by several factors that are not typically implicated in the positivity effect in attention and memory, including social network (Golden et al., 2009; Litwin and Shiovitz-Ezra, 2010), health/disabilities (Fonseca et al., 2014), and prosocial behavior (Aknin et al., 2013). Future research needs to examine differences and similarities in the underlying mechanisms between the age-related changes in well-being and the age-related positivity effect in attention and memory.

There are also other important limitations of this study. First, we had a relatively large difference in baseline age across participants. While this allowed us to estimate a growth curve from middle- to old age, it may have introduced a potential confound across those in different age groups. The large baseline age differences also posed an analytic challenge in examining short-term potential reciprocal effects between cognitive control and well-being. While there is a substantial body of work on the reciprocal interaction between well-being and cognitive function (e.g., Boyle et al., 2010; Allerhand et al., 2014; Lewis et al., 2017), these large-scale studies primarily concerned American or West European samples. Future similar studies on different cultures are critical for understanding whether age, cognitive control, and well-being interact similarly or differently depending on individuals’ backgrounds.

Second, our sample size was relatively small and limited to females. Although the small sample size is compromised by the relatively large number of data points we had (*n* = 382; Curran et al., 2010), a large number of assessments we administered per participant (*n* = 12) resulted in a high attrition rate, which may have resulted in biased estimates. Furthermore, previous research has documented sex differences in well-being (Pinquart and Sörensen, 2001) as well as in cognitive control (Tun and Lachman, 2008). Females also experience age-related changes that are not observed in males (e.g., menopause), which can affect cognitive control abilities (Sakaki and Mather, 2012; Herrera et al., 2017). Future research, therefore, needs to examine whether our results will be replicated in a bigger sample, and systematically address whether females and males show similar interactions between age and cognitive control abilities. Given general difficulties in maintaining older adult participants in longitudinal studies (Bonk, 2010), such future research may need to consider using a smaller number of assessments per participant to ensure reasonable statistical power in a cost-effective manner.

Third, while our study found a steady increase in well-being in old age, all of our participants were healthy adults. In contrast, cognitive control abilities show a rapid decline in proximity to death (Johansson et al., 2004; MacDonald et al., 2011). Well-being is also known to rapidly decrease in proximity to death (Gerstorf et al., 2010). Furthermore, we did not include a formal screening test for dementia or MCI. Further studies need to consider the effects of systematic dysfunction associated with death as well as dementia. Fourth, the current study focused on only one source of individual differences that modulate age-related changes in well-being—cognitive control. Given that previous studies suggest that other individual differences in goals also play a role in the cultural differences in age-related changes in emotional experiences (Fung et al., 2010), future research should take into account the effects of these additional variables to fully understand how age affects individuals’ well-being across different cultures.

## Data Availability Statement

The datasets generated for this study are available on request to the corresponding author.

## Ethics Statement

The studies involving human participants were reviewed and approved by Institutional Review Board of the Tohoku University Graduate School of Medicine. The patients/participants provided their written informed consent to participate in this study.

## Author Contributions

AY, RN, MS, KM, and RK designed the study. RN and RK collected data. AY, MS, and KM performed analyses. AY and MS wrote an initial draft. All authors approved the final version of the manuscript.

## Conflict of Interest

This study was based on an industry–academic collaboration at the Tohoku University (https//www.sairct.idac.tohoku.ac.jp/smart-ageing-international-research-center/square/) and supported by Curves Japan Co., Ltd. However, the funding source had no involvement in the study design, data collection, data analysis, the interpretation of data, and writing up the manuscript.

## References

[B1] Aknin L. B. Barrington-Leigh C. P. Dunn E. W. Helliwell J. F. Burns J. Biswas-Diener R.. (2013). Prosocial spending and well-being: cross-cultural evidence for a psychological universal. J. Pers. Soc. Psychol. 104, 635–652. 10.1037/a0031578 23421360

[B2] Allerhand M. Gale C. R. Deary I. J. (2014). The dynamic relationship between cognitive function and positive well-being in older people: a prospective study using the English Longitudinal Study of Aging. Psychol. Aging 29, 306–318. 10.1037/a0036551 24955999 PMC4196750

[B3] Boehm J. K. Kubzansky L. D. (2012). The heart’s content: the association between positive psychological well-being and cardiovascular health. Psychol. Bull. 138, 655–691. 10.1037/a0027448 22506752

[B4] Bonk J. (2010). A road map for the recruitment and retention of older adult participants for longitudinal studies. J. Am. Geriatr. Soc. 58, S303–S307. 10.1111/j.1532-5415.2010.02937.x 21029058

[B5] Boyle P. A. Buchman A. S. Barnes L. L. Bennett D. A. (2010). Effect of a purpose in life on risk of incident alzheimer disease and mild cognitive impairment in community-dwelling older personslife purpose and AD risk. JAMA Psychiatry 67, 304–310. 10.1001/archgenpsychiatry.2009.208 20194831 PMC2897172

[B6] Carstensen L. L. DeLiema M. (2018). The positivity effect: a negativity bias in youth fades with age. Curr. Opin. Behav. Sci. 19, 7–12. 10.1016/j.cobeha.2017.07.009 30327789 PMC6186441

[B7] Carstensen L. L. Turan B. Scheibe S. Ram N. Ersner-Hershfield H. Samanez-Larkin G. R.. (2011). Emotional experience improves with age: evidence based on over 10 years of experience sampling. Psychol. Aging 26, 21–33. 10.1037/a0021285 20973600 PMC3332527

[B8] Chapman K. R. Bing-Canar H. Alosco M. L. Steinberg E. G. Martin B. Chaisson C.. (2016). Mini mental state examination and logical memory scores for entry into Alzheimer’s disease trials. Alzheimers Res. Ther. 8:9. 10.1186/s13195-016-0176-z 26899835 PMC4762168

[B9] Charles S. T. Luong G. Almeida D. M. Ryff C. Sturm M. Love G. (2010). Fewer ups and downs: daily stressors mediate age differences in negative affect. J. Gerontol. B Psychol. Sci. Soc. Sci. 65B, 279–286. 10.1093/geronb/gbq002 20123699 PMC2981451

[B10] Charles S. T. Reynolds C. A. Gatz M. (2001). Age-related differences and change in positive and negative affect over 23 years. J. Pers. Soc. Psychol. 80, 136–151. 10.1037/0022-3514.80.1.136 11195886

[B11] Curran P. J. Obeidat K. Losardo D. (2010). Twelve frequently asked questions about growth curve modeling. J. Cogn. Dev. 11, 121–136. 10.1080/15248371003699969 21743795 PMC3131138

[B12] Davis J. Marra C. Najafzadeh M. Liu-Ambrose T. (2010). The independent contribution of executive functions to health related quality of life in older women. BMC Geriat. 10:16. 10.1186/1471-2318-10-16 20359355 PMC2867806

[B13] Enkvist Å. Ekström H. Elmståhl S. (2013). Associations between cognitive abilities and life satisfaction in the oldest-old. Results from the longitudinal population study Good Aging in Skåne. Clin. Interv. Aging 8, 845–853. 10.2147/cia.s45382 23874091 PMC3712740

[B14] Fjell A. M. Sneve M. H. Grydeland H. Storsve A. B. Walhovd K. B. (2016). The disconnected brain and executive function decline in aging. Cereb. Cortex 27, 2303–2317. 10.1093/cercor/bhw082 27073220

[B15] Fonseca R. Kapteyn A. Lee J. Zamarro G. Feeney K. (2014). A longitudinal study of well-being of older europeans: does retirement matter? J. Popul. Ageing 7, 21–41. 10.1007/s12062-014-9094-7 24729798 PMC3979480

[B16] Fung H. H. (2013). Aging in culture. Gerontologist 53, 369–377. 10.1093/geront/gnt024 23585454

[B17] Fung H. H. Carstensen L. L. (2006). Goals change when life’s fragility is primed: lessons learned from older adults, the September 11 attacks and sars. Soc. Cogn. 24, 248–278. 10.1521/soco.2006.24.3.248

[B18] Fung H. H. Isaacowitz D. M. Lu A. Y. Li T. (2010). Interdependent self-construal moderates the age-related negativity reduction effect in memory and visual attention. Psychol. Aging 25, 321–329. 10.1037/a0019079 20545417

[B19] Fung H. H. Isaacowitz D. M. Lu A. Y. Wadlinger H. A. Goren D. Wilson H. R. (2008a). Age-related positivity enhancement is not universal: older chinese look away from positive stimuli. Psychol. Aging 23, 440–446. 10.1037//0882-7974.23.2.440 18573017

[B20] Fung H. H. Stoeber F. S. Yeung D. Y.-L. Lang F. R. (2008b). Cultural specificity of socioemotional selectivity: age differences in social network composition among germans and hong kong chinese. J. Gerontol. B Psychol. Sci. Soc. Sci. 63, P156–P164. 10.1093/geronb/63.3.p156 18559680

[B21] Gale C. R. Cooper R. Craig L. Elliott J. Kuh D. Richards M.. (2012). Cognitive function in childhood and lifetime cognitive change in relation to mental wellbeing in four cohorts of older people. PLoS One 7:e44860. 10.1371/journal.pone.0044860 22970320 PMC3438162

[B22] Gana K. Saada Y. Amieva H. (2015). Does positive affect change in old age? Results from a 22-year longitudinal study. Psychol. Aging 30, 172–179. 10.1037/a0038418 25436598

[B23] Gerstorf D. Ram N. Mayraz G. Hidajat M. Lindenberger U. Wagner G. G.. (2010). Late-life decline in well-being across adulthood in germany, the united kingdom and the united states: something is seriously wrong at the end of life. Psychol. Aging 25, 477–485. 10.1037/a0017543 20545432 PMC2975938

[B24] Godin J. Armstrong J. J. Wallace L. Rockwood K. Andrew M. K. (2018). The impact of frailty and cognitive impairment on quality of life: employment and social context matter. Int. Psychogeriatr. 31, 789–797. 10.1017/S1041610218001710 30421692

[B25] Golden J. Conroy R. M. Bruce I. Denihan A. Greene E. Kirby M.. (2009). Loneliness, social support networks, mood and wellbeing in community-dwelling elderly. Int. J. Geriatr. Psychiatry 24, 694–700. 10.1002/gps.2181 19274642

[B26] Grossmann I. Karasawa M. Kan C. Kitayama S. (2014). A cultural perspective on emotional experiences across the life span. Emotion 14, 679–692. 10.1037/a0036041 24749641

[B27] Hakoda Y. Sasaki M. (1990). Group version of the stroop and reverse-stroop test: the effects of reaction mode, order and practice. Jpn. J. Educ. Psychol. 38, 389–394. 10.5926/jjep1953.38.4_389

[B28] Herrera A. Y. Hodis H. N. Mack W. J. Mather M. (2017). Estradiol therapy after menopause mitigates effects of stress on cortisol and working memory. J. Clin. Endocrinol. Metab. 102, 4457–4466. 10.1210/jc.2017-00825 29106594 PMC5718702

[B29] Isaacowitz D. M. Smith J. (2003). Positive and negative affect in very old age. J. Gerontol. B Psychol. Sci. Soc. Sci. 58, P143–P152. 10.1093/geronb/58.3.p143 12730307

[B30] Jivraj S. Nazroo J. Vanhoutte B. Chandola T. (2014). Aging and subjective well-being in later life. J. Gerontol. B Psychol. Sci. Soc. Sci. 69, 930–941. 10.1093/geronb/gbu006 24569002 PMC4296137

[B31] Johansson B. Hofer S. M. Allaire J. C. Maldonado-Molina M. M. Piccinin A. M. Berg S.. (2004). Change in cognitive capabilities in the oldest old: the effects of proximity to death in genetically related individuals over a 6-year period. Psychol. Aging 19, 145–156. 10.1037/0882-7974.19.1.145 15065938

[B32] Knight M. Seymour T. L. Gaunt J. T. Baker C. Nesmith K. Mather M. (2007). Aging and goal-directed emotional attention: distraction reverses emotional biases. Emotion 7, 705–714. 10.1037/1528-3542.7.4.705 18039037

[B33] Ko S. G. Lee T. H. Yoon H. Y. Kwon J. H. Mather M. (2011). How does context affect assessments of facial emotion? The role of culture and age. Psychol. Aging 26, 48–59. 10.1037/a0020222 21038967 PMC3062682

[B34] Kwon Y. Scheibe S. Samanez-Larkin G. R. Tsai J. L. Carstensen L. L. (2009). Replicating the positivity effect in picture memory in Koreans: evidence for cross-cultural generalizability. Psychol. Aging 24, 748–754. 10.1037/a0016054 19739932 PMC2775417

[B35] Lang F. R. Carstensen L. L. (2002). Time counts: future time perspective, goals, and social relationships. Psychol. Aging 17, 125–139. 10.1037/0882-7974.17.1.125 11931281

[B36] Lewis N. A. Turiano N. A. Payne B. R. Hill P. L. (2017). Purpose in life and cognitive functioning in adulthood. Neuropsychol. Dev. Cogn. B Aging Neuropsychol. Cogn. 24, 662–671. 10.1080/13825585.2016.1251549 27819520

[B37] Litwin H. Shiovitz-Ezra S. (2010). Social network type and subjective well-being in a national sample of older americans. Gerontologist 51, 379–388. 10.1093/geront/gnq094 21097553 PMC3095651

[B38] MacDonald S. W. S. Hultsch D. F. Dixon R. A. (2011). Aging and the shape of cognitive change before death: terminal decline or terminal drop? J. Gerontol. B Psychol. Sci. Soc. Sci. 66B, 292–301. 10.1093/geronb/gbr001 21300703 PMC3078759

[B39] Mather M. Carstensen L. L. (2003). Aging and attentional biases for emotional faces. Psychol. Sci. 14, 409–415. 10.1111/1467-9280.01455 12930469

[B40] Mather M. Carstensen L. L. (2005). Aging and motivated cognition: the positivity effect in attention and memory. Trends Cogn. Sci. 9, 496–502. 10.1016/j.tics.2005.08.005 16154382

[B41] Mather M. Knight M. (2005). Goal-directed memory: the role of cognitive control in older adults’ emotional memory. Psychol. Aging 20, 554–570. 10.1037/0882-7974.20.4.554 16420131

[B42] Matsuoka K. Uno M. Kasai K. Koyama K. Kim Y. (2006). Estimation of premorbid IQ in individuals with Alzheimer’s disease using Japanese ideographic script (Kanji) compound words: japanese version of national adult reading test. Psychiatry Clin. Neurosci. 60, 332–339. 10.1111/j.1440-1819.2006.01510.x 16732750

[B43] Milham M. P. Erickson K. I. Banich M. T. Kramer A. F. Webb A. Wszalek T.. (2002). Attentional control in the aging brain: insights from an fMRI study of the stroop task. Brain Cogn. 49, 277–296. 10.1006/brcg.2001.1501 12139955

[B44] Ono Y. Yoshimura K. (2001). WHO SUBI Manual. Tokyo: Kanekoshobo.

[B45] Opitz P. C. Gross J. J. Urry H. L. (2012). Selection, optimization and compensation in the domain of emotion regulation: applications to adolescence, older age, and major depressive disorder. Soc. Pers. Psychol. Compass 6, 142–155. 10.1111/j.1751-9004.2011.00413.x

[B46] Petrican R. Moscovitch M. Schimmack U. (2008). Cognitive resources, valence, and memory retrieval of emotional events in older adults. Psychol. Aging 23, 585–594. 10.1037/a0013176 18808248

[B47] Pinquart M. Sörensen S. (2001). Gender differences in self-concept and psychological well-being in old age: a meta-analysis. J. Gerontol. B Psychol. Sci. Soc. Sci. 56, P195–P213. 10.1093/geronb/56.4.p195 11445606

[B48] Raghavan N. Samtani M. N. Farnum M. Yang E. Novak G. Grundman M.. (2013). The ADAS-Cog revisited: novel composite scales based on ADAS-Cog to improve efficiency in MCI and early AD trials. Alzheimers Dement. 9, S21–S31. 10.1016/j.jalz.2012.05.2187 23127469 PMC3732822

[B49] R-Core-Team (2017). R: A Language and Environment for Statistical Computing. Vienna, Austria: R Foundation for Statistical Computing Available online at: https://www.R-project.org/.

[B50] Reed A. E. Chan L. Mikels J. A. (2014). Meta-analysis of the age-related positivity effect: age differences in preferences for positive over negative information. Psychol. Aging 29, 1–15. 10.1037/a0035194 24660792

[B51] Riediger M. Schmiedek F. Wagner G. G. Lindenberger U. (2009). Seeking pleasure and seeking pain: differences in prohedonic and contra-hedonic motivation from adolescence to old age. Psychol. Sci. 20, 1529–1535. 10.1111/j.1467-9280.2009.02473.x 19891749

[B52] Ryff C. D. (1989). Happiness is everything, or is it? Explorations on the meaning of psychological well-being. J. Pers. Soc. Psychol. 57, 1069–1081. 10.1037/0022-3514.57.6.1069

[B53] Sakaki M. Mather M. (2012). How reward and emotional stimuli induce different reactions across the menstrual cycle. Soc. Pers. Psychol. Compass 6, 1–17. 10.1111/j.1751-9004.2011.00415.x 22737180 PMC3380631

[B54] Sakaki M. Nga L. Mather M. (2013). Amygdala functional connectivity with medial prefrontal cortex at rest predicts the positivity effect in older adults’ memory. J. Cogn. Neurosci. 25, 1206–1224. 10.1162/jocn_a_00392 23530897 PMC4104303

[B55] Sakaki M. Raw J. Findlay J. Thottam M. (2019). Advanced aging enhances the positivity effect in memory: due to cognitive control or age-related decline in emotional processing? Collabra Psychol. 5:49 10.1525/collabra.222

[B56] Scheibe S. Carstensen L. L. (2010). Emotional aging: recent findings and future trends. J. Gerontol. B Psychol. Sci. Soc. Sci. 65B, 135–144. 10.1093/geronb/gbp132 20054013 PMC2821944

[B57] Sell H. Nagpal R. (1992). Assessment of Subjective Well-Being: The Subjective Well-Being Inventory (SUBI). New Delhi: World Health Organization, Regional Office for South-East Asia.

[B58] Steptoe A. Wardle J. (2011). Positive affect measured using ecological momentary assessment and survival in older men and women. Proc. Natl. Acad. Sci. U S A 108, 18244–18248. 10.1073/pnas.1110892108 22042845 PMC3215003

[B59] Steptoe A. Deaton A. Stone A. A. (2015). Subjective wellbeing, health, and ageing. Lancet 385, 640–648. 10.1016/s0140-6736(13)61489-0 25468152 PMC4339610

[B60] Takeuchi H. Taki Y. Hashizume H. Sassa Y. Nagase T. Nouchi R.. (2011). Effects of training of processing speed on neural systems. J. Neurosci. 31, 12139–12148. 10.1523/JNEUROSCI.2948-11.2011 21865456 PMC6623215

[B61] Tsai J. L. (2017). Ideal affect in daily life: implications for affective experience, health, and social behavior. Curr. Opin. Psychol. 17, 118–128. 10.1016/j.copsyc.2017.07.004 28950957 PMC5659332

[B62] Tsai J. L. Miao F. F. Seppala E. (2007). Good feelings in christianity and buddhism: religious differences in ideal affect. Pers. Soc. Psychol. Bull. 33, 409–421. 10.1177/0146167206296107 17312321

[B63] Tucker-Drob E. M. (2011). Neurocognitive functions and everyday functions change together in old age. Neuropsychology 25, 368–377. 10.1037/a0022348 21417532 PMC3086995

[B64] Tun P. A. Lachman M. E. (2008). Age differences in reaction time and attention in a national telephone sample of adults: education, sex, and task complexity matter. Dev. Psychol. 44, 1421–1429. 10.1037/a0012845 18793073 PMC2586814

[B65] Urry H. L. Gross J. J. (2010). Emotion regulation in older age. Curr. Dir. Psychol. Sci. 19, 352–357. 10.1177/0963721410388395

[B66] Wang J. He L. Jia L. Tian J. Benson V. (2015). The ‘positive effect’ is present in older chinese adults: evidence from an eye tracking study. PLoS One 10:e0121372. 10.1371/journal.pone.0121372. 25880585 PMC4400038

[B67] Wechsler D. (1987). Manual for the Wechsler Memory Scale-Revised. San Antonio, TX: Psychological Corporation.

[B68] Wechsler D. (1997). Wechsler Adult Intelligence Scale—III. San Antonio, TX: The Psychological Corporation.

[B69] Wilson R. S. Boyle P. A. Segawa E. Yu L. Begeny C. T. Anagnos S. E.. (2013). The influence of cognitive decline on well-being in old age. Psychol. Aging 28, 304–313. 10.1037/a0031196007 23421323 PMC3692607

[B70] You J. Fung H. H. L. Isaacowitz D. M. (2009). Age differences in dispositional optimism: a cross-cultural study. Eur. J. Ageing 6:247. 10.1007/s10433-009-0130-z 28798608 PMC5547345

