# Supplementary material for: The Role of Cognitive Control in Age-Related Changes in Well-Being
Source: Front Aging Neurosci. 2020 Jul 9;12:198. doi: 10.3389/fnagi.2020.00198 (PMC7396630; doi:10.3389/fnagi.2020.00198)
Supplement: Supplementary file 1 [file Data_Sheet_1.pdf]

Supplemental Materials

Details of Cognitive Measures

All cognitive tests were administered in a group format, which included up to six participants as done in previous studies (Nouchi, Kobayashi, Nouchi, & Kawashima, 2019; Takeuchi et al., 2011; Takeuchi et al., 2015). Participants were allocated separate desks and chairs and were not allowed to talk to each other before and during the test. Before each test, the experimenters provided participants with the instructions and addressed any questions from participants. Once all participants fully understood the task procedure, the experimenters asked them to start the task. After each task, the experimenters collected completed test sheets and distributed those for the next test.

Stroop. During the Stroop task, participants completed the two versions (both were based on paper and pencil): Stroop 1 and Stroop 2 (Hakoda & Sasaki, 1990; Takeuchi et al., 2011). In the Stroop task 1, participants were shown a list of color words ("black", "red", "yellow", "blue", and "green") printed in black with five color patches with different colors (black, red, yellow, blue, and green); their task was to indicate the color of the word by selecting an appropriate patch from the five patches. In the Stroop task 2, participants were shown the same list of color words printed in a color which does not match the word (e.g., "blue" in red ink) with the five color patches (same as those used in

the Stroop task 1); participants were asked to select the color patch consistent with the word ignoring the ink color. In both tasks, participants were asked to select the correct patches as many as possible in 1 min. We obtained the Stroop interference score by subtracting accuracy in the Stroop task 2 minus accuracy in the Stroop task 1 divided by the accuracy in the Stroop task 1.

Digit forward/backward. During the digit forward/backward task, the tester read aloud a series of number sequences and asked participants to write down the numbers in forward order or revere order. The digit forward had 16 sequences from 2 digits and 9 digits (2 sequences in each). The digit backward had 14 sequences from 2 digits to 8 digits (2 sequences in each). For the digit forward/backward task, we used the number of correct answers.

Japanese Reading Test (JART). The Japanese Reading Test (JART) is a Japanese version of the National Adult Reading Test (NART; Nelson, 1982) and concerns individuals' vocabulary (Matsuoka, Uno, Kasai, Koyama, & Kim, 2006). JART includes 25 words based on Chinese characters (e.g. 親父, 煙草). The stimuli were shown on a paper in a randomized order. The participants were asked to read each word and write down how to read it using Japanese Hiragana characters. The primary measure for this task was the number of correct items.

Verbal memory. The verbal memory is a subtest of the Wechsler Memory Scale Revised (Wechsler, 1987). The tester read aloud a short story and participants were asked to memorize the story while listening to it. Immediately after the story, participants were asked to write down the story. The story was scored in terms of the number of story units recalled, as specified in the WMS-R scoring protocol.

Other Cognitive Measures

In addition to the tasks described in text, participants completed the Digit Cancellation Task (D-CAT) which concerns attention (Takeshi Hatta et al., 2004) and digit symbol-coding, and symbol search from the WAIS (Wechsler, 1997).

Digit Cancellation Task (D-CAT). The Digit Cancellation Task (D-CAT) evaluated attention (T. Hatta, Ito, & Yoshizaki, 2000). The test sheet consisted of 12 rows of 50 digits. Each row contains five sets of numbers 0–9 arranged in random order. Consequently, any one digit appeared five times in each row with randomly determined neighbors. D-CAT comprised three such sheets. Participants were instructed to search for target number(s) that had been specified to them and to delete each one with a slash mark as quickly and as accurately as possible until the experimenter sends a stop signal. Three trials were used, first with a single target number (6), second with two target numbers (9 and 4), and third with three (8, 3, and 7). Each trial was given for 1 min.

Consequently, the total time required for D-CAT was 3 min. In the second and third trials, it was emphasized that all the target numbers instructed should be cancelled without omission. The primary measure of this test was the number of hits (correct answers). We used only the number of hits in the first trial.

Digit symbol coding. The Digit Symbol Coding is a subtest of WAIS-III (Wechsler, 1997) and concerns processing speed. Participants are shown symbol-digit pairs and a list of digits. Their task is to write down the corresponding symbol as fast as possible. The primary measure of this test was the number of correct answers.

Symbol search. The Symbol Search (SS) task is also a subtest of WAIS-III (Wechsler, 1997) and considered to measure processing speed. The task includes 60 items. Participants visually scan two groups of symbols (a target group and a search group) and report whether either of the target symbols matched any symbol in the search group. Participants respond as many items as possible within a 120 s time limit. The primary measure of this test is the number of correct answers.

Other Self-Reported Questionnaires

Other questionnaires administered in the study included the Generalized Self-Efficacy Scale (Narita et al., 1995; Schwarzer & Jerusalem, 1995); the Kikuchi's Scale of Social Skills (Kikuchi, 2004); the Center for Epidemiology Studies Depression

Scale (Radloff, 1977; Shima, 1988); the Emotion Regulation Questionnaire (Gross & John, 2003; Yoshizu, Sekiguchi, & Amemiya, 2013), the Self-Esteem Scale (Rosenberg, 1965; Yamamoto, Matsui, & Yamanari, 1982), the Everyday Attentional Experiences Questionnaire (Shinohara, Yamada, Kanda, & Usui, 2007), the Behavioral Inhibition/Behavioral Activation Scales (Carver & White, 1994; Takahashi et al., 2007), the Effortful Control Scale (Rothbart, Ahadi, & Evans, 2000; Yamagata, Takahashi, Shigemasu, Ono, & Kijima, 2005), the General Health Questionnaire-12 (Goldberg, 1978; Nakagawa & Daibo, 1985), the Emotional Intelligence Scale (Uchiyama, Shimai, Utsuki, & Otake, 2001), NEO-FFI (Costa & McCrae, 2010; Shimonaka, Nakazato, Gondo, & Takayama, 1999), the revised UCLA Loneliness Scale (Moroi, 1991; Russell, Peplau, & Cutrona, 1980).

Details of the Growth Curve Models

R codes used to perform the main growth curve modeling analysis are described below:

When the dependent variable is mental health:

lmer(mental_health ~ cognitive_control + age + age*cognitive_control + age^2 + age^2*cognitive_control + (1 + age | subject), data=data)

When the dependent variable is mental fatigue:

lmer(mental_fatigue ~ cognitive_control + age + age*cognitive_control + age^2 + age^2*cognitive_control + (1 + age + age^2 | subject), data=data)

Supplemental Results

To account for the effects of possible dementia/MCI cases, we created another composite measure based on the Wechsler logical memory and vocabulary performance, both of which is typically correlated with performance in standard screening batteries for dementia (Chapman et al., 2016; Raghavan et al., 2013). We ran the same analyses as those described in the main text while adding this additional composite score on general cognitive decline associated with dementia or MCI as a covariate; due to the conversion errors, we omitted some random effects. The results from these additional analyses are largely consistent with those from our analyses without this covariate, suggesting that the effects of cognitive control we observed are not necessarily driven by the effects of dementia or MCI (S-Table 1).

S-Table 1 Results from multilevel growth curve analyses on two aspects of subjective well-being after controlling for general cognitive decline with age

	Mental health			Mental fatigue		
Fixed effects	Coefficient		*p*	Coefficient		*p*
Intercept	-0.05		0.731	-0.08		0.571
Cognitive control	0.09		0.648	-0.16		0.345
General cognitive decline	0.27		0.110	-0.22		0.121
Age	0.33	*	0.018	-0.43	*	0.001
Age*Cognitive control	-0.32		0.066	0.26	*	0.026
Age^2	0.04		0.601	0.15	*	0.002
Age^2*Cognitive control	-0.04		0.646	-0.11	*	0.017
Random effects	Variance			Variance		
Intercept	0.64			0.67		
Age	0.10			NA		
Age^2	NA			0.01		

References

Carver, C. S., & White, T. L. (1994). Behavioral inhibition, behavioral activation, and affective responses to impending reward and punishment: The BIS/BAS Scales. *Journal of Personality and Social Psychology, 67*(2), 319-333. doi:10.1037/0022-3514.67.2.319

Chapman, K. R., Bing-Canar, H., Alosco, M. L., Steinberg, E. G., Martin, B., Chaisson, C., . . . Stern, R. A. (2016). Mini Mental State Examination and Logical Memory scores for entry into Alzheimer’s disease trials. *Alzheimer's Research and Therapy, 8*(1), 9. doi:10.1186/s13195-016-0176-z

Costa, P., & McCrae, R. R. (2010). The NEO Personality Inventory: 3. *Odessa, FL: Psychological assessment resources*.

Goldberg, D. (1978). *Manual of the general health questionnaire*. Windsor: NFER.

Gross, J. J., & John, O. P. (2003). Individual differences in two emotion regulation processes: Implications for affect, relationships, and well-being. *Journal of Personality and Social Psychology, 85*(2), 348-362. doi:10.1037/0022-3514.85.2.348

Hakoda, Y., & Sasaki, M. (1990). Group version of the stroop and reverse-stroop test : The effects of reaction mode, order and practice. *The Japanese journal of educational psychology., 38*(4), p389-394.

Hatta, T., Ito, Y., & Yoshizaki, K. (2000). *D-CAT manual (Screening test for attention)*. Osaka.: Union Press.

Hatta, T., Masui, T., Ito, Y., Ito, E., Hasegawa, Y., & Matsuyama, Y. (2004). Relation Between the Prefrontal Cortex and Cerebro-Cerebellar Functions: Evidence From the Results of Stabilometrical Indexes. *Applied Neuropsychology, 11*(3), 153-160. doi:10.1207/s15324826an1103_3

Kikuchi, A. (2004). Notes on the Researches Using KiSS-18. *Bulletin of the Faculty of Social Welfare, Iwate Prefectural University, 6*(2), 41-51.

Matsuoka, K., Uno, M., Kasai, K., Koyama, K., & Kim, Y. (2006). Estimation of premorbid IQ in individuals with Alzheimer's disease using Japanese ideographic script (Kanji) compound words: Japanese version of National Adult Reading Test. *Psychiatry Clin Neurosci, 60*(3), 332-339. doi:10.1111/j.1440-1819.2006.01510.x

Moroi, K. (1991). Dimensions of the revised UCLA Loneliness Scale. *Studies in humanities.*(42), p23-51. doi:info:doi/10.14945/00003890

Nakagawa, Y., & Daibo, I. (1985). *Manual for the Japanese version of the GHQ.* Tokyo: Nihon Bunka Kagakusha.

Narita, K., Shimonaka, Y., Nakazato, K., Kawaai, C., Sato, S., & Osada, Y. (1995). A japanese version of the generalized self-efficacy scale: Scale utility from the life span perspective. *The Japanese Journal of Educational Psychology, 43*(3), 306-314. doi:10.5926/jjep1953.43.3_306

Nelson, H. E. (1982). *National Adult Reading Test (NART)*. Windsor, UK: NFER-Nelson.

Nouchi, R., Kobayashi, A., Nouchi, H., & Kawashima, R. (2019). Newly Developed TV-Based Cognitive Training Games Improve Car Driving Skills, Cognitive Functions, and Mood in Healthy Older Adults: Evidence From a Randomized Controlled Trial. *Frontiers in Aging Neuroscience, 11*(99). doi:10.3389/fnagi.2019.00099

Radloff, L. S. (1977). The CES-D scale: A self-report depression scale for research in the general population. *Applied Psychological Measurement, 1*(3), 385-401. doi:10.1177/014662167700100306

Raghavan, N., Samtani, M. N., Farnum, M., Yang, E., Novak, G., Grundman, M., . . . DiBernardo, A. (2013). The ADAS-Cog revisited: Novel composite scales based on ADAS-Cog to improve efficiency in MCI and early AD trials. *Alzheimer's & Dementia, 9*(1, Supplement), S21-S31. doi:https://doi.org/10.1016/j.jalz.2012.05.2187

Rosenberg, M. (1965). *Society and the adolescent self-image*: Princeton University Press.

Rothbart, M. K., Ahadi, S. A., & Evans, D. E. (2000). Temperament and personality: origins and outcomes. *Journal of Personality and Social Psychology, 78*(1), 122-135.

Russell, D., Peplau, L. A., & Cutrona, C. E. (1980). The revised UCLA Loneliness Scale: Concurrent and discriminant validity evidence. *Journal of Personality and Social Psychology, 39*(3), 472-480. doi:10.1037/0022-3514.39.3.472

Schwarzer, R., & Jerusalem, M. (1995). Generalized Self-Efficacy scale. In J. Weinman, S. Wright, & M. Johnston (Eds.), *Measures in health psychology: A user's portfolio. Causal and control beliefs* (pp. 35-37). Windsor, UK: NFER-NELSON.

Shima, S. (1988). *Guidance for using CES-D scale*. Japan: Chiba Test Center.

Shimonaka, Y., Nakazato, K., Gondo, Y., & Takayama, M. (1999). *NEO-PI-R, NEO-FFI manual for the Japanese Version.* Tokyo: Tokyo Shinri Inc.

Shinohara, K., Yamada, N., Kanda, K., & Usui, S. (2007). Attentional experiences in daily life and individual differences in subjective mental workload ratings. *The Japanese journal of ergonomics, 43*(4), 201-211. doi:10.5100/jje.43.201

Takahashi, Y., Yamagata, S., Kijima, N., Shigemasu, K., Ono, Y., & Ando, J. (2007).

Gray's Temperament Model: Development of Japanese Version of BIS/BAS Scales and A Behavior Genetic Investigation Using the Twin Method. *The Japanese Journal of Personality, 15*(3), 276-289. doi:10.2132/personality.15.276

Takeuchi, H., Taki, Y., Hashizume, H., Sassa, Y., Nagase, T., Nouchi, R., & Kawashima, R. (2011). Effects of Training of Processing Speed on Neural Systems. *The Journal of Neuroscience, 31*(34), 12139-12148. doi:10.1523/jneurosci.2948-11.2011

Takeuchi, H., Taki, Y., Nouchi, R., Hashizume, H., Sekiguchi, A., Kotozaki, Y., . . . Kawashima, R. (2015). Working memory training impacts the mean diffusivity in the dopaminergic system. *Brain Structure and Function, 220*(6), 3101-3111. doi:10.1007/s00429-014-0845-2

Uchiyama, K., Shimai, S., Utsuki, N., & Otake, K. (2001). *EQS Manual (in Japanese).* Tokyo, Japan: Jitsumukyoiku Syuppan.

Wechsler, D. (1987). Manual for the Wechsler memory scale-revised. *San Antonio, TX: Psychological Corporation*.

Wechsler, D. (1997). *Wechsler Adult Intelligence Scale Third edition.* San Antonio, TX: The Psychological Corporation.

Yamagata, S., Takahashi, Y., Shigemasu, K., Ono, Y., & Kijima, N. (2005). Development and Validation of Japanese Version of Effortful Control Scale for Adults. *The Japanese Journal of Personality, 14*(1), 30-41. doi:10.2132/personality.14.30

Yamamoto, M., Matsui, Y., & Yamanari, Y. (1982). The Structure of Perceived Aspects of Self. *Japanese Journal of Educational Psychology, 30*, 64-68.

Yoshizu, J., Sekiguchi, R., & Amemiya, T. (2013). Development of a Japanese version of Emotion Regulation Questionnaire. *The japanese journal of research on emotions, 20*(2), 56-62. doi:10.4092/jsre.20.56
